# Driver Head–Hand Cooperative Action Recognition Based on FMCW Millimeter-Wave Radar and Deep Learning

**DOI:** 10.3390/s25082399

**Published:** 2025-04-10

**Authors:** Lianlong Zhang, Xiaodong Chen, Zexin Chen, Jiawen Zheng, Yinliang Diao

**Affiliations:** College of Electronic Engineering, South China Agricultural University, Guangzhou 510642, China; lianlongzhang@stu.scau.edu.cn (L.Z.); cxdehcom@stu.scau.edu.cn (X.C.); zexinchen@stu.scau.edu.cn (Z.C.); zheng_jiawen@stu.scau.edu.cn (J.Z.)

**Keywords:** millimeter-wave radar, driver behavior detection, micro-Doppler spectrum, deep learning

## Abstract

Driver status plays a critical role in ensuring driving safety. However, the current visual recognition-based methods for detecting driver actions and status are often limited to factors such as ambient light condition, occlusion, and privacy concerns. In contrast, millimeter-wave radar offers various advantages such as high accuracy, ease of integration, insensitivity to light condition, and low cost; therefore, it has been widely used for monitoring vital signals and in action recognition. Despite this, the existing studies on driver action recognition have been hindered by limited accuracy and a narrow range of detectable actions. In this study, we utilized a 77 GHz millimeter-wave frequency-modulated continuous-wave radar to construct a dataset encompassing seven types of driver head–hand cooperative actions. Furthermore, a deep learning network model based on VGG16-LSTM-CBAM using micro-Doppler spectrograms as input was developed for action classification. The experimental results demonstrated that, compared to the existing CNN-LSTM and ALEXNET-LSTM networks, the proposed network achieves a classification accuracy of 99.16%, effectively improving driver action detection.

## 1. Introduction

The rapid advancement of smart solutions and information technology has revolutionized modern transportation systems [1]. With the rise of artificial intelligence, intelligent driving systems have also developed rapidly [2]. Vehicle systems now incorporate sensor-based interaction designs, such as visual, tactile, auditory, and body-sensing modalities, to assist drivers by monitoring hand gestures, voice, posture, and other vital signals, ensuring safety and convenience during driving. Drowsy driving remains one of the leading causes of road accidents; therefore, the intelligent detection of driver actions and status is essential in ensuring driving safety [3].

Currently, research has been conducted on the detection of driver head or body motion [4,5,6,7,8,9], as well as driver status [10,11,12], with a heavy reliance on visible light sensors and computer vision techniques. While these sensors provide high resolution, they face significant limitations, such as sensitivity to lighting conditions, background noise, and potential privacy concerns, particularly in public vehicles [13,14]. Infrared sensors provide high spatial resolution and functionality in low-light conditions, making them suitable for detecting nighttime pedestrian targets. However, for in-vehicle applications, their use is underexplored, as they remain susceptible to occlusion, lighting variations, and ambient heat—such as from seat heating devices—often reducing accuracy for complex actions. Furthermore, near-infrared imaging inherits privacy concerns by producing identifiable images.

Millimeter-wave radar has proven to be a valuable alternative due to its high detection accuracy, insensitivity to lighting conditions, ease of integration, and low cost. It has become widely adopted in biometric signal detection [15,16,17,18,19,20,21] and action and posture recognition [22,23,24,25,26,27,28]. It has proven effective in a variety of occupational and domestic scenarios. For example, Bresnahan et al. [29] applied millimeter-wave radar to detect deep tendon reflexes, creating spectrograms for the rapid and accurate analysis of patients’ reflex status. Arab et al. [30] used a 24 GHz millimeter-wave radar to sample and analyze eight different human body movements, applying a dual-branch CNN model to classify the actions, achieving a classification accuracy of 98.85%.

In the context of vehicle driver assistance, the use of millimeter-wave radar for driver status detection and action recognition is being continuously explored, thereby promoting safe driving [31]. In [32], frequency-modulated continuous-wave (FMCW) radar and CNN models were utilized to recognize and classify four types of head movements of a driver for abnormal behavior detection. A fine-tuned CNN model was employed to classify and recognize fatigue-related actions, with breathing signals under different human body states collected via millimeter-wave radar to analyze whether the driver was fatigued [33]. In [34], FMCW radar was used to collect six types of head movement signals to measure the distance Doppler and Doppler spectrograms during head and neck movements, proving the feasibility of using a radar to monitor the driver’s head movements. In [35], four different head movement signals from the driver were collected by installing millimeter-wave radar on the steering wheel; the action signals were classified using CNN networks. In [36], millimeter-wave radar was installed on the car dashboard to collect eight common head movement signals, which were then classified and recognized using a Deep-CNN model. However, these studies have limitations, focusing mainly on head motions and overlooking the cooperative movements of the head and hands, which are critical for accurately analyzing driver behavior in real-world situations.

In this study, we used millimeter-wave FMCW radar to collect micro-Doppler features from combined head and hand motions, creating a dataset of seven head–hand actions recorded from five volunteers. We also proposed a novel deep learning network VGG16-LSTM-CBAM, which integrated the VGG16 backbone with LSTM and CBAM attention blocks to enhance classification accuracy. The experimental results demonstrated the effectiveness of our proposed network by comparing it with other common deep learning models. The paper is organized as follows: Section 2 introduces the background theory of FMCW radar, Section 3 introduces the network model used in this experiment, and Section 4 presents the dataset. Section 5 discusses the action recognition accuracy under different networks, with conclusions drawn in Section 6.

## 2. Related Theories

### 2.1. Millimeter-Wave Radar Echo Model

In the FMCW radar system, a sequence of signals is transmitted whose frequency changes linearly with a function of time. The transmitted signal of the FMCW radar can be represented as follows:

(1) $$s\left(t\right)=A_{T}cos(2\pi f_{c}t+\pi \frac{B}{T}t^{2}+\phi_{0})$$

where $A_{T}$ is the amplitude of the transmitted signal, $f_{c}$ is the radar carrier frequency, T is the pulse interval, B is the bandwidth, and $\phi_{0}$ is the initial phase of the chirp.

The corresponding received echo signal is represented as follows:

(2) $$s\left(t\right)=A_{R}\cos\left(2\pi f_{c}(t-\tau )+\pi \frac{B}{T}{\left(t-\tau \right)}^{2}+\varphi_{0}\right)$$

where $A_{R}$ is the amplitude of the echo signal, τ=2r/c is the frequency shift caused by the distance between the radar and the target, and c is the speed of light.

Then, the transmitted and received signals pass through the frequency mixer, and high and low-frequency signals are generated, which is represented as follows:

(3) $$S_{IF}\left(t\right)=A_{IF}\cos(2\pi f_{c}\tau +\pi \frac{B}{T}(2t-\tau )\tau )=A_{IF}\cos\left(2\pi f_{c}\tau +2\pi \frac{B}{T}\tau -\pi \frac{B}{T}\tau^{2}\right)$$

where $A_{IF}$ is the difference frequency signal amplitude.

In practical applications, when the radar-detected target exhibits radial motion, the frequency of the received signal undergoes an additional frequency shift due to the Doppler effect. This phenomenon originates from the compression or stretching of the wavefront that electromagnetic waves experience during reflection from a moving target. Specifically, if the target moves with a velocity v relative to the radar, its echo signal will carry a frequency offset $∆f_{d}$ that is proportional to the velocity. This Doppler frequency shift can be expressed as follows:

(4) $$∆f_{d}=\frac{2v}{\lambda }=\frac{2vf_{c}}{c}$$

where λ represents the wavelength of the transmitted signal, and c is the speed of light.

### 2.2. Micro-Doppler Spectrogram Acquisition

The Doppler shift described in Equation (4) applies to scenarios where the target moves uniformly as a whole. However, when there are local micro-motions in the target (such as human joint movements), its echo will be superimposed with periodic time-varying modulation components. The Doppler shift caused by such micro-motions exhibits dynamic harmonic characteristics, known as the micro-Doppler effect [37], which need to be extracted through joint time–frequency analysis. The Short-Time Fourier Transform (STFT) is a commonly used joint time–frequency analysis method for analyzing non-stationary signals, and it is also the time–frequency analysis method employed in this paper to extract micro-Doppler features. It is expressed as follows:

(5) $$S(\omega ,\tau )=F\left(x\left(t\right)\cdot w\left(t-\tau \right)\right)=\int_{-\infty }^{+\infty }x(t)\cdot w(t-\tau )e^{-j\omega t}dt$$

where x(t) represents the echo signal containing micro-Doppler modulation, w(t) is the window function, and ω is the angular frequency.

**Figure 1:**
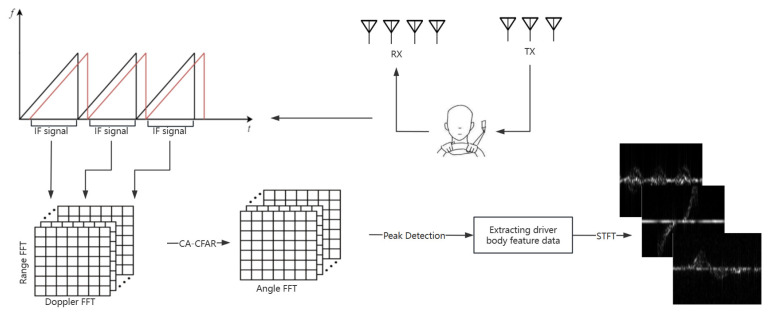
Radar signal processing flowchart. In the upper left chart, the black curves represent the transmitted signals, and the red curves depict the received signals.

As shown in Figure 1, to obtain the micro-Doppler features of the driver’s actions, the following steps were performed: First, the radar echo signals were windowed along the fast-time dimension, followed by a distance-Fast Fourier Transform (FFT). The actual distance of the target was obtained based on the peak frequency component in the spectrum and the radar range resolution. Next, an FFT was applied along the slow-time dimension to obtain the distance-Doppler spectrum features. Subsequently, the Cell Averaging–Constant False Alarm Rate (CA-CFAR) algorithm [38] and angle-FFT were used to generate a range–angle heatmap. The Density-Based Spatial Clustering of Applications with Noise (DBSCAN) clustering algorithm was applied to extract the main motion areas of the driver, and the STFT was used to capture the time-domain information of the signal sequence. Finally, the time–frequency representation containing the driver’s action micro-Doppler features was obtained.

## 3. VGG16-LSTM-CBAM Network

**Figure 2:**
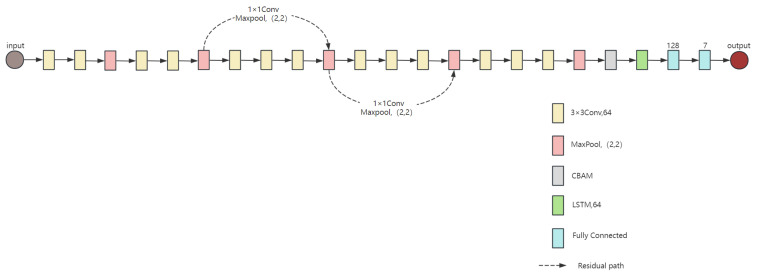
VGG16-LSTM-CBAM network model.

In image recognition, various deep learning network models have been proposed, each with different capabilities for handling image classification and recognition tasks. In the dataset described in previous section, micro-Doppler feature spectrograms were generated, allowing for the application of image recognition network architectures. The proposed network structure is shown in Figure 2. This network is primarily based on the VGG16 module and modified to include 13 convolutional and 2 fully connected layers, a Long Short-Term Memory (LSTM) module, and a Convolutional Block Attention Module (CBAM) attention module. Additionally, the network also includes multiple max-pooling layers and linear rectification function (ReLU) activation function layers.

### 3.1. VGG16-LSTM

The VGG network model was proposed by the Visual Geometry Group at the University of Oxford in 2014 [39]. Since its introduction, several versions of the model have been developed. The VGG16 model, as its name suggests, primarily consists of 16 convolutional layers. VGG16 uses many convolution kernels of size 3 × 3, which enables the network to extract finer image features with a smaller receptive field. This design helps minimize feature loss during pooling and enhances feature extraction. The proposed network model is built upon the VGG16 model, incorporating module improvements.

After passing through the VGG16 network, the 3D tensor is flattened into a 2D tensor feature sequence and input into the LSTM network, which is known for the effective capture of hidden features and information in images. A SoftMax layer is used at the end of the network to classify and recognize the results from the fused VGG16-LSTM network, ultimately providing the predicted target. Additionally, residual connections are added to prevent overfitting caused by the deeper network layers [40]. Moreover, the network remains focused on low-level features, which helps to maintain or even improve performance after the modification.

### 3.2. CBAM

To help VGG16 capture more accurate key information in image processing and analysis, an attention mechanism module was also introduced into the network. This allowed the model to focus more on the critical parts of the input data during decision-making thus improving training accuracy and efficiency. The attention mechanism module added here is the CBAM, which combines channel attention and spatial attention to enhance the learning ability of the network. These two modules are connected serially; the input feature map first passes through the channel attention module to obtain a corrected feature map, which is then processed by the spatial attention module, resulting in an output feature map enhanced by CBAM.

**Figure 3:**
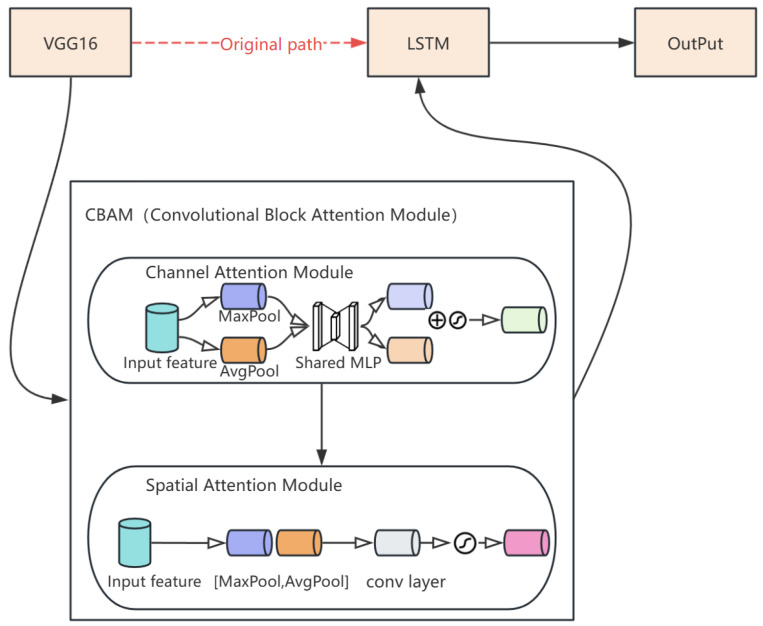
Schematic diagram of VGG-LSTM with CBAM.

As shown in Figure 3, the CBAM attention mechanism is introduced between VGG16 and LSTM networks, allowing the model to focus on more detailed feature information and ignore irrelevant data, thereby improving classification accuracy. In the VGG16-LSTM network, the crucial process is the feature fusion and transmission between the two networks. Therefore, the CBAM attention mechanism is placed between these networks, acting as a bridge for feature fusion and information transmission. This ensures that the features extracted by VGG16 are thoroughly filtered and integrated before being passed into the LSTM network for further feature extraction and behavior prediction. This design helps the network improve prediction accuracy without increasing its depth.

## 4. Dataset

### 4.1. Experimental Setup

**Figure 4:**
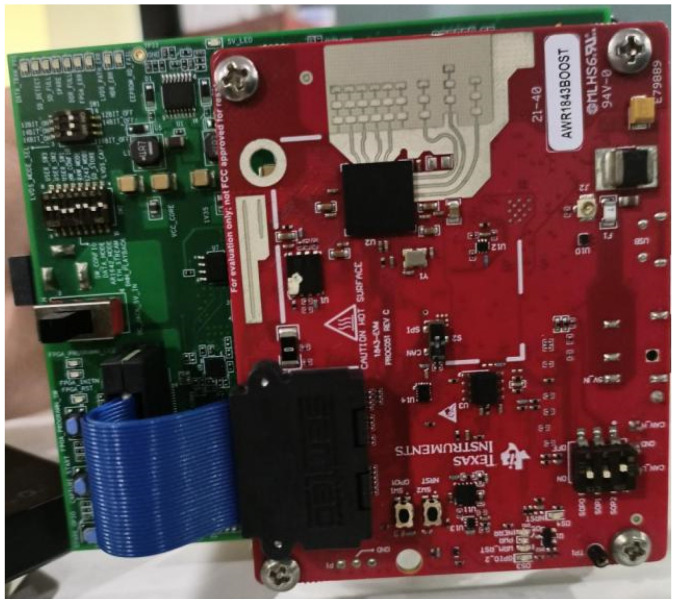
Experimental setup: connection between the AWR1843BOOST radar sensor and the DCA1000EVM data acquisition board.

The main equipment for this experiment consisted of the AWR1843BOOST millimeter-wave radar sensor [41] and the DCA1000EVM [42] data acquisition board from Texas Instruments. This device was used to collect driver action signals and generate micro-Doppler spectrograms as the experimental dataset. The AWR1843BOOST radar sensor had three transmitting antennas and four receiving antennas, and it was connected to the DCA1000EVM acquisition board, as shown in Figure 4.

### 4.2. Safety of Millimeter-Wave Radar

According to the specifications of the AWR1843BOOST millimeter-wave radar development board, under the typical operating conditions (3 transmit channels active with 4 receive channels; 25 ms frame interval), the total power consumption remains below ~2.0 W. Complementary measurements using the DCA1000EVM data capture card (operating at 5V DC supply with typical current draw of 700 mA) demonstrate an additional 3.5 W power requirement for real-time IF signal acquisition. This results in a combined system power budget of <6 W for simultaneous radar sensing and raw data streaming. Additionally, the total RF output power is 12 dBm (approximately 15.8 milliwatts). The horizontal 3 dB beam width is ±28°, and the elevation 3 dB beam width is ±14°. The skin penetration depth of the 77 GHz electromagnetic wave is only about 0.3 mm. In an extreme case where the electromagnetic wave is entirely transmitted into human tissue without any reflection, the absorbed power density at a distance of 20 cm from the antenna is estimated to be 0.8 W/m^2^. This value is significantly lower than the basic limit of 20 W/m^2^ set by the International Commission on Non-Ionizing Radiation Protection (ICNIRP) for the general public, thereby complying with health and safety regulations.

### 4.3. Data Acquisition

**Figure 5:**
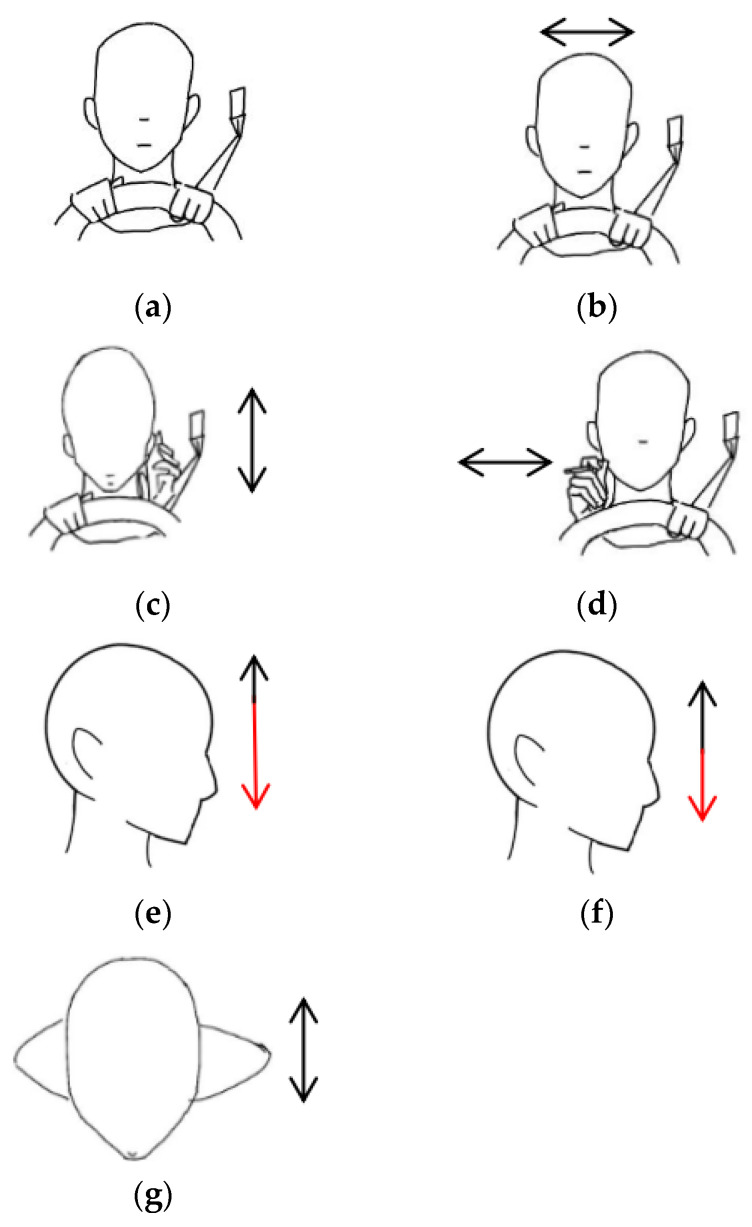
Schematic diagram of different actions, the images are as follows: (**a**) stationary; (**b**) head turning; (**c**) hand raised to ear; (**d**) hand raised to mouth; (**e**) head drooping forward; (**f**) head nodding; (**g**) leaning forward. Red arrows are used to emphasize the extent of the downward head movement.

**Figure 6:**
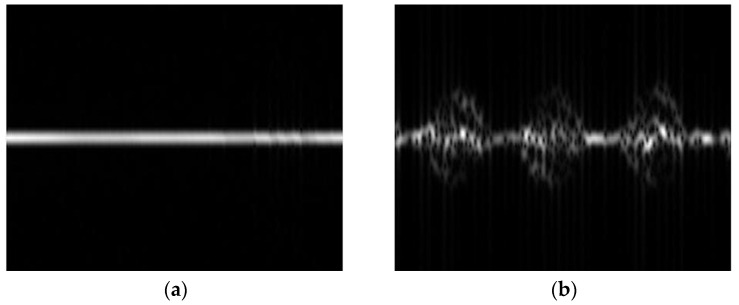

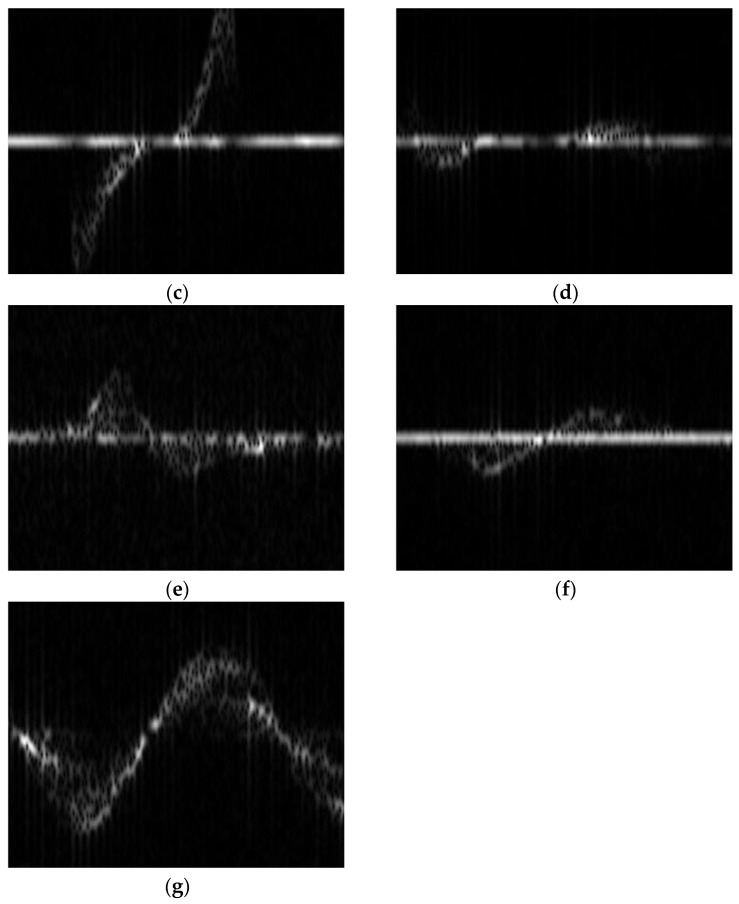
The micro-Doppler spectrograms of different actions, the images are as follows: (**a**) stationary; (**b**) head turning; (**c**) hand raised to ear; (**d**) hand raised to mouth; (**e**) head drooping forward; (**f**) head nodding; (**g**) leaning forward.

The driver action dataset including the head and hand movements are illustrated in Figure 5, which presents the following seven different driving scenarios: stationary, head turning (left–right), hand raised to ear (phone conversation), hand raised to mouth (smoking), head drooping forward (drowsiness), head nodding, and leaning forward (hard braking). The drowsiness action primarily focuses on head movement, such as the driver’s head drooping or nodding rapidly, leading to vertical shaking or trembling [43]. Nodding, on the other hand, involves a steady up-and-down motion of the head, which distinguishes it from the drowsiness action. The smoking action is designed to capture the driver’s hand movement by simulating the motion of bringing a cigarette to the mouth. The phone call action is designed to capture the driver raising a phone to their ear. The time–frequency spectrograms for each action are shown in Figure 6. To simulate a real driving scenario, during data collection, the subject sat on a chair facing forward, with the radar placed about 0.7 m to the right front of the target. The radar parameters are shown in Table 1.

In total, 5 participants participated in the experiment, including three males and two females, aged between 22 and 26, all with driving experience. Each participant performed each action 20 times, resulting in 700 data samples. Five-fold cross-validation was used to divide the collected data into five subsets. Each subset was used as the test set once, and the other four subsets were used for training. This process was repeated five times, and the average cross-validation accuracy was used as the model evaluation metric.

## 5. Experimental Results and Analysis

### 5.1. Performance Analysis

**Figure 7:**
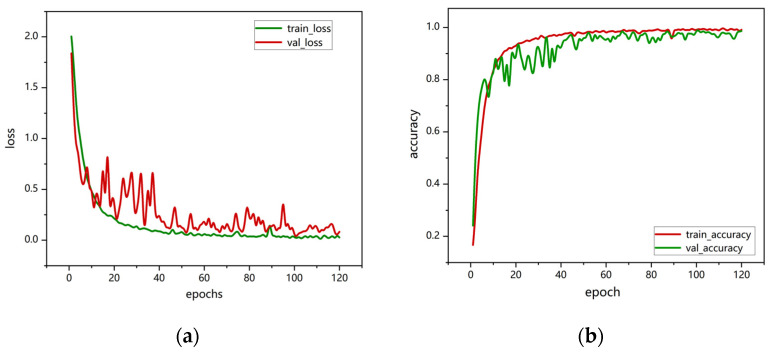
VGG16-LSTM-CBAM training and validation performance: (**a**) loss curve; (**b**) accuracy graph.

The model training was conducted on an GeForce RTX 4090D (Nvidia, Santa Clara, CA, USA). The hyperparameters in the deep learning network are set as follows: batch size of 8, cross-entropy loss function, and 120 epochs for training. The performance analysis is based on accuracy and confusion matrices, as shown in Figure 7. As the number of training epochs increased, accuracy gradually improved. During the first 30 training iterations, the accuracy fluctuated. After 40 epochs, the model converged, and both training and validation accuracies stabilized at around 99%.

**Figure 8:**
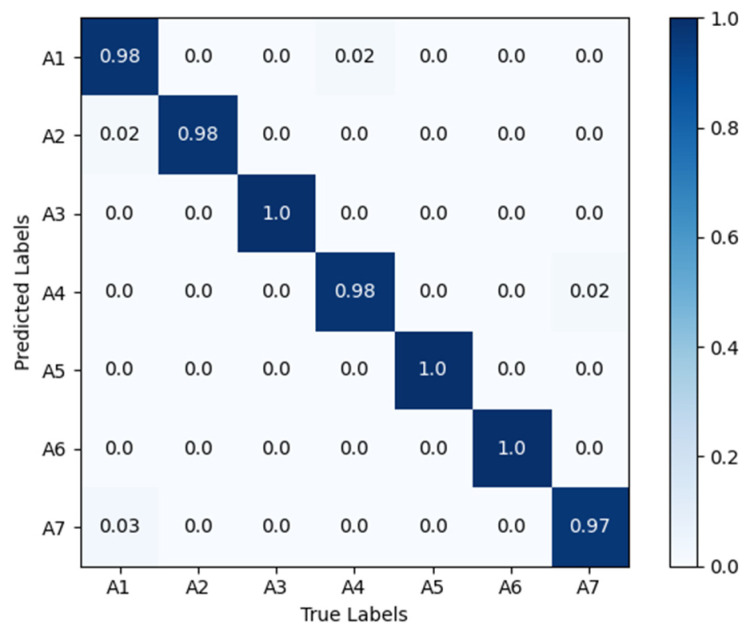
Confusion matrix diagrams for VGG16-LSTM-CBAM. A1 to A7 respectively denote: head nodding; head drooping forward; head turning; leaning forward; stationary; hand raised to ear; hand raised to mouth.

Figure 8 shows the confusion matrix diagrams for VGG16-LSTM-CBAM, in the matrix, the rows represent the true action sequences, and the columns represent the action sequences recognized by the algorithm. A1 to A7 respectively denote: head nodding; head drooping forward; head turning; leaning forward; stationary; hand raised to ear; hand raised to mouth. The results reveal that, in the VGG16-LSTM-CBAM network, the best performance was achieved for “head turning”, “stationary”, and “hand raised to ear”, all reaching 100% accuracy. However, there are slight errors in recognizing “nodding” and “drowsiness”, as these two actions are quite similar in behavior. Additionally, there were some misclassifications for “smoking” and “hard braking”.

### 5.2. Comparison with Other Networks

**Figure 9:**
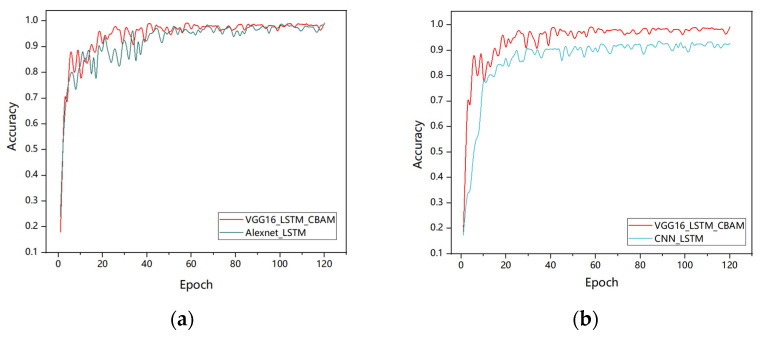

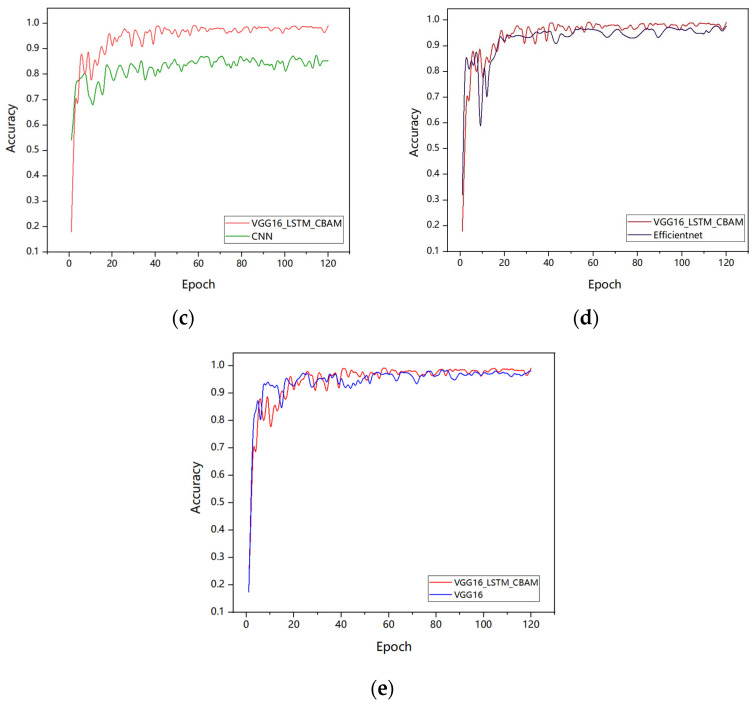
Comparison of test accuracy between VGG16_LSTM_CBAM and the different networks performance: (**a**) Alexnet-LSTM; (**b**) CNN-LSTM; (**c**) CNN; (**d**) EfficientNetB0 and (**e**) VGG16.

Figure 9 illustrates the training accuracy trends of six models, namely CNN, CNN-LSTM, AlexNet-LSTM, EfficientNetB0, traditional VGG16, and VGG16-LSTM-CBAM. As shown in the figure, the training accuracy of all six networks improves with increasing iteration counts. On the same dataset, the VGG16-LSTM-CBAM network achieves an accuracy of 99.16%, representing a 6.45% improvement over the traditional CNN-LSTM and 13.73% over the baseline CNN. Notably, the traditional VGG16, whose fully connected layers consist of two 4096-dimensional dense layers, contributes over 90% of the total network parameters. In this study, we adopt a dimensionality collapse strategy to replace the original dual 4096-dimensional layers with a single 64-dimensional lightweight structure. Additionally, batch normalization (BN) layers are incorporated after each convolutional block to accelerate training convergence and mitigate overfitting. The final model achieves a 95% reduction in parameter count compared to the traditional VGG16 while improving accuracy by 1.12% (detailed in Table 2), conclusively demonstrating the superior performance of the proposed network.

**Figure 10:**
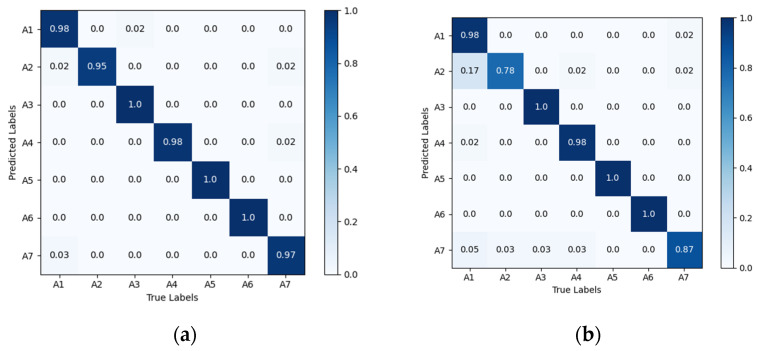

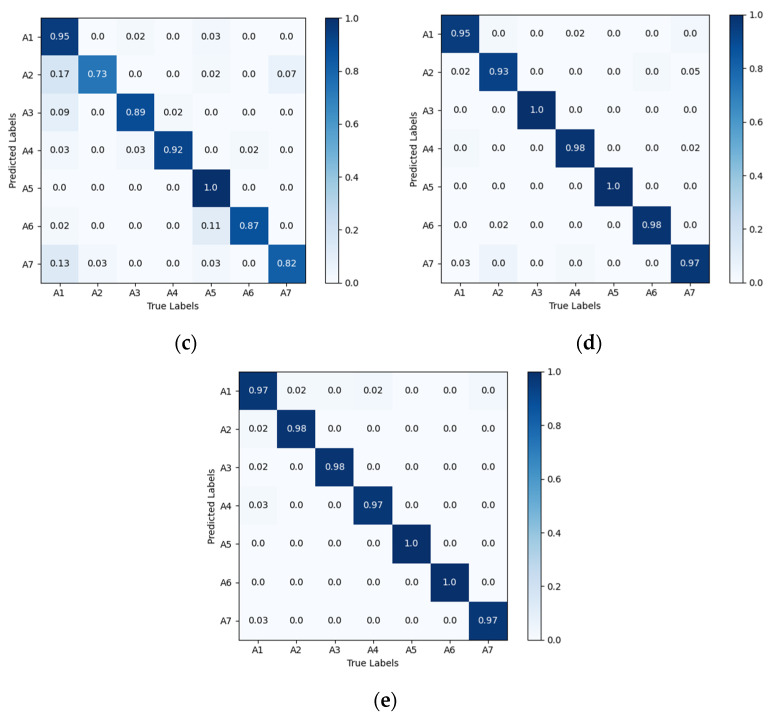
Confusion matrix diagrams for different networks performance: (**a**) Alexnet-LSTM; (**b**) CNN-LSTM; (**c**) CNN; (**d**) EfficientNetB0 and (**e**) VGG16.

Comparison of the confusion matrix diagrams for different networks is shown in Figure 10. As seen, compared to the Alexnet-LSTM, CNN-LSTM, CNN, EfficientNetB0, and VGG16 networks, the proposed VGG16-LSTM-CBAM network demonstrates a smaller discrepancy between the recognition results and the actual action sequences, achieving higher recognition accuracy. This further validates the feasibility and superiority of the method proposed in this paper for driver head–hand cooperative action recognition.

To evaluate the contribution of the modules proposed in the VGG16-LSTM-CBAM model, we further conducted four ablation experiments on our dataset (see Table 3). The baseline VGG16 model (with two 4096-dimensional fully connected (FC) layers) achieved an accuracy of 98.04%. Incorporating the LSTM temporal module enables the model to capture the sequential dependencies in input data, improving accuracy to 98.12%, while increasing the mode size slightly. The original FC-4096 layer contains over 90% of the model’s total parameters. By replacing it with an FC-64 layer, this model not only reduces computational load but also effectively inhibits overfitting risks, aligning with the lightweight concept, reducing mode size by 95%, increased accuracy by 0.56%, resulting in an accuracy of 99.16% for our final model.

## 6. Conclusions

In this study, we utilized an FMCW radar to construct a dataset encompassing seven types of driver’s head–hand cooperative actions. A deep learning network model based on VGG16-LSTM-CBAM using micro-Doppler spectrograms as input was developed for action classification. The experimental results demonstrated that, compared to the existing CNN-LSTM, EfficientNetB0, and ALEXNET-LSTM networks, the proposed network achieves a classification accuracy of 99.16%, effectively improving the driver actions detection and thus demonstrating the practical reference value for real-world applications. However, the human actions designed for this experiment were relatively fixed and simple. In real-world scenarios, more complex actions and interference may occur, which will increase the difficulty of detection. Therefore, future research will explore the inclusion of more complex driver actions and further recognition of facial features.

## Figures and Tables

**Table 1:** TI AWR1843 FMCW millimeter-wave radar configuration parameter.

Parameters	Value
Starting frequency/GHz	77
Effective bandwidth/MHz	2667
Chirp samples/count	128
Sampling frequency/ksps	4000
Slope/(MHz/μs)	21
Frame chirp count/count	255
Frame period/ms	270

**Table 2:** Comparison of different networks.

Network	Accuracy	Recall	F1 Score	Parameter Count (M)	FLOPs (G)	Model Size (MB)
VGG16-LSTM-CBAM	99.16%	99.13%	99.15%	16.25	91.00	61.99
VGG16	98.04%	97.94%	97.99%	304.17	90.37	1160.33
Alexnet-LSTM	98.03%	98.00%	98.02%	4.99	6.10	19.03
EfficientNetB0	97.76%	98.12%	97.86%	4.13	0.78	15.76
CNN-LSTM	92.71%	92.50%	92.61%	0.25	0.08	0.94
CNN	85.43%	84.80%	85.10%	0.30	0.03	1.16

**Table 3:** Ablation experiments.

Network	Accuracy	F1 Score	Model Size (MB)
VGG16 only	98.04%	97.99%	1160.33
+LSTM	98.12%	98.05%	1160.89
Modified FC	98.60%	98.63%	61.46
VGG16-LSTM-CBAM	99.16%	99.15%	61.99

## Data Availability

The data used in this paper are collected through our own experiments and are not yet publicly available. However, data may be obtained from the authors upon reasonable request.
